# Effect of Heating and Massaging of Meibomian Glands on Their Imaging

**DOI:** 10.3390/medicina60101603

**Published:** 2024-09-29

**Authors:** Justin E. Pettayil, Samya Haque, Mohammed Fardin, Sandeep Kaur Dhallu, Sònia Travé-Huarte, James S. Wolffsohn, Debarun Dutta

**Affiliations:** 1 Optometry and Vision Sciences Research Group, Aston University, Birmingham AL10 9AB, UK190097644@aston.ac.uk (S.H.); 200044714@aston.ac.uk (M.F.); s.trave-huarte@aston.ac.uk (S.T.-H.); d.dutta@aston.ac.uk (D.D.); 2 Department of Clinical, Pharmaceutical and Biological Sciences, University of Hertfordshire, Hatfield AL10 9EU, UK

**Keywords:** meibomian glands, dry eye, tear film, heating, eyelid massage

## Abstract

*Background and Objectives:* Infrared light is used to image the Meibomian glands through their thermal profile. This study aimed to investigate the effects of a combination of heating and an eyelid massage on Meibomian gland visibility and tear film parameters. *Materials and Methods:* Twenty-four participants (26 ± 6.9 years) were enrolled in this prospective study, which involved imaging the Meibomian glands of both the lower and upper eyelid and assessing the non-invasive breakup time (NIBUT), tear meniscus height (TMH), and blink rate (using the CA-800, Topcon) at baseline after five minutes of eyelid warming followed by a five-minute eyelid massage. The second session, which was randomised in sequence, repeated the same measurements but without the inclusion of any eyelid warming or massage as the control condition. *Results:* While there was no change in lower lid Meibomian gland appearance as a result of eyelid heating, eyelid massage, or multiple lid eversion (median 2.0, range 0.0 to 4.0; *p* = 0.782), there was a change in upper lid appearance 5 min after heating and lid massage (*p* = 0.025), but again, multiple lid eversion had no effect (*p* > 0.05). The NIBUT decreased on second lid eversion (*p* = 0.049), although this was not evident on the third lid eversion (*p* = 0.090). The effect on NIBUT was also apparent with heating (*p* = 0.034 immediately after) but was sustained with 5 min of eyelid massage (*p* = 0.031). The TMH increased with heating (*p* < 0.001), and this effect was sustained with 5 min of eyelid massage (*p* = 0.011), but there was no lid eversion effect (*p* > 0.05). The blink rate was unaffected by heating, eyelid massage, or multiple eversions of the eyelids (median 24 blinks/min, range 8 to 59 blinks/min; *p* = 0.61). Conclusions: Eyelid warming can increase the visibility of the Meibomian glands, although this effect was only observed with upper lid imaging and the effect dissipated after 5 min of eyelid massage. Warming and massage also disrupt the tear film, as does multiple lid eversion, emphasising the need to use the least invasive tear film assessment techniques first.

## 1. Introduction

The Meibomian glands (MGs) are superficial sebaceous glands located in the tarsal plates. In healthy individuals, their orifices are regularly distributed along the lid margins, with 25–40 located on the upper lid and 20–30 on the lower lid [1]. MGs secrete meibum, which combines with the aqueous layer on the ocular surface to maintain tear film stability [2]. Meibomian gland dysfunction (MGD) is a chronic condition affecting the MGs that can be identified by blockages in the terminal ducts or variations in the quality or number of glandular secretions. A recent systematic review revealed that a strikingly high prevalence of MGD was reported in various prospective clinical studies, with prevalence reaching 41.7% across all ages, 47.8% in elderly people, and 35.7% in paediatric or young adult populations [3]. MGD can lead to alterations in the tear film stability, eye irritation, noticeable clinical or sub-clinical inflammation, and ocular surface disease. Management of MGD typically involves eyelid warming to liquify pathologically altered meibum, followed by an eyelid massage, which allows the removal of obstructive materials from gland openings and orifices [4,5]. An eyelid massage might include manual manipulation, electric toothbrush-like devices used by a clinician in-office, or forcible expression procedures, which usually require the use of an anaesthetic [4,6]. Various other treatments are used to enhance meibum liquification of pathologically solidified meibum and increase expression, and these treatments include thermal pulsation [7], intense pulsed light treatment [8], Tixel [9] and low-level light therapy [10].

Warm compresses applied for between five and fifteen minutes are widely used as a mainstay treatment for MGD, followed by brief eyelid hygiene (cleaning the eyelashes) between one and four times a day. Applying a warm compress heats the eyelids, softening the waxy meibum, melting the obstructing meibum, and allowing it to blend into the tear film to aid tear spread and reduce tear evaporation [11]. Although a warm compress results in an increase in the quality of meibum expression, patients with short or partial glands may not fully benefit from this treatment [12]. In addition, non-compliance with the lid-warming treatment regime often undermines the treatment outcome [11]. After warm compresses have been applied, lid scrubs and therapeutic expression are recommended to remove debris from the lid margin and facilitate the release of meibum that may block the MG orifices [13,14]. 

Imaging of MG or meibography has been used for over 40 years [15]. It is a technique that allows in vivo observation of the glands, which is particularly important for understanding the health of the MGs and monitoring the management of MGD. Contrast, sharpness, and field of view have seen substantial enhancements in the imaging of MGs [16], while imaging artefacts have been notably reduced. To quantify the visibility of MGs captured in a greyscale image, various metrics have been used, such as energy, relative energy, entropy, standard deviation irregularity, mean pixel intensity, standard deviation, median pixel intensity, mode pixel intensity, and kurtosis and skewness of a histogram [15,17]. An absence of MGs is believed to indicate ‘dropout’ of MGs, which may directly correlate with MGD and dry eye disease (DED) signs and symptoms. Research involving patients undergoing isotretinoin treatment indicates that changes in the reflectivity or contrast of MGs might serve as a measure of gland functionality [18]. This is evidenced by the decrease in reflectivity during the treatment phase, followed by an increase after the treatment is discontinued [18]. However, meibography uses infrared light which detects the thermal signature of the glands which may be affected by heating or mechanical manipulation, such as massage or the act of lid eversion needed to expose the palpebral surface of the eyelids. Therefore, this study examined the effects of combined heating and lid massage on MG imaging along with tear film parameters evaluation techniques to assess their agreement.

## 2. Materials and Methods

All procedures were conducted at the Aston Eye Clinic according to the Helsinki Declaration for Experimentation on Humans, and the study was approved by the Aston University Research Ethics Committee. Prior to commencement, all participants provided written informed consent, after they were given ample time to review and comprehend the Participant Information Sheet. 

### 2.1. Participants

Participants over 18 years of age were included in the study; there were no restrictions related to wearing habitual contact lenses, although participants diagnosed with a history of any ocular disease within the last 6 months and unilateral best corrected visual acuity below 6/12 were excluded. Any participant using any medication that could affect the ocular surface or tearing was excluded. Experienced contact lens wearers were asked to remove their lenses before starting the assessments and could wear lenses after the completion of the tests.

### 2.2. Protocol Design

The study involved two visits for each participant: one incorporating interventions and the other serving as a control, separated by an interval of two days to three weeks. At the initial visit, meibography and tear film assessments were performed at baseline, after 5 min of eyelid warming and again after 5 min of eyelid massaging. During the subsequent visit, meibography was performed three times at five-minute intervals without any intervention to determine whether the eyelid everting alone impacted the glands and tear film assessments.

### 2.3. Intervention

**Figure 1:**
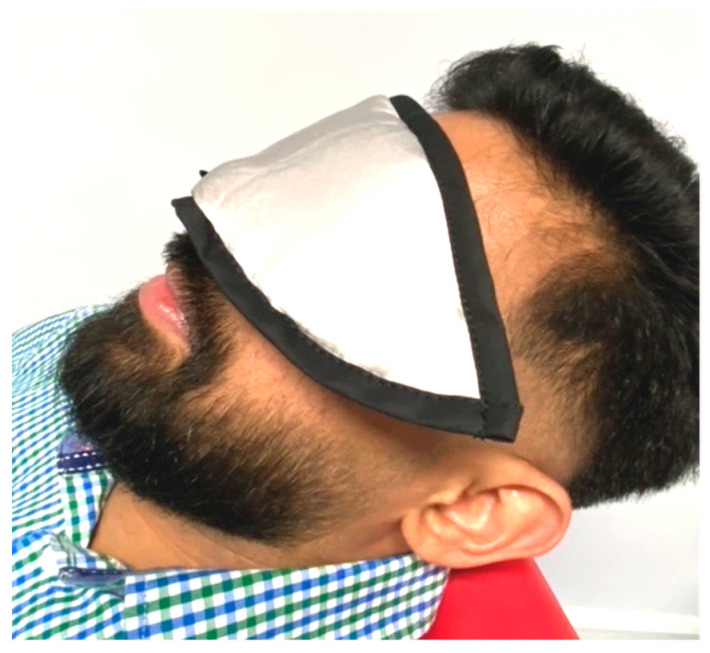
Representative photograph of the Blepha EyeBag used by a participant.

Eyelid warming was facilitated using a Blepha EyeBag^®^ (Figure 1), which was heated in a microwave for 30 s at a power level of 800 W as per the manufacturer’s instructions. The manual eyelid massage involved gentle manipulation using the index and middle fingers for 5 min, aligning with the gland directions.

### 2.4. Measurements

**Figure 2:**
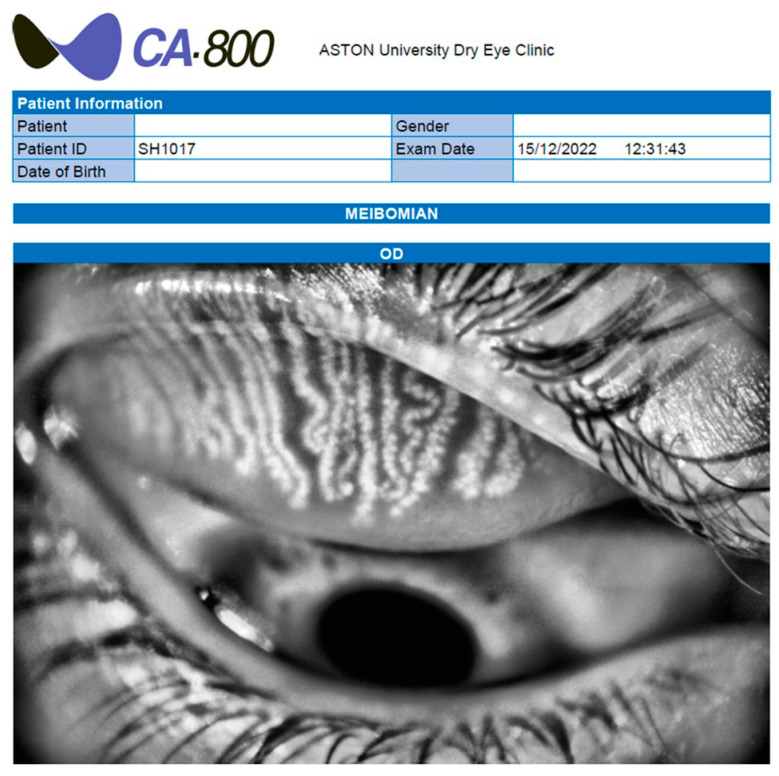
A representative photograph of the CA-800 Corneal Analyzer used for the study with imaged meibomian glands.

Infrared meibography and tear film parameter assessments were conducted using the CA-800 Corneal Analyzer (Figure 2; Topcon, Tokyo, Japan) [19]. All participants completed the Ocular Surface Disease Index (OSDI) questionnaire at the start. The CA-800 Corneal Analyzer was used to obtain rate measurements automatically without participants’ awareness to prevent voluntary blinking and potential measurement interference. Additionally, the tear meniscus height (TMH) was measured while participants maintained primary gaze (three measurements at the lower lid margin beneath the pupil), and the non-invasive tear breakup time (NIBUT) was recorded (as an average of three measurements) objectively. MG images were captured following upper and inferior eyelid eversion [15], with clear images selected for grading. To grade the MGs, the percentage of the eyelid that displays total and/or partial acinar loss [12] was evaluated by a masked observer with the Meiboscale [20].

### 2.5. Statistical Analysis

The data were analysed using IBM SPSS Statistics (v29, Chicago, IL, USA). Normal distribution was established with the Kolmogorov–Smirnov test. As all of the data were found to be statistically significantly different from a normal distribution (*p* < 0.05), the data between visits were compared with related samples Friedman’s two-way analysis of variance by ranks. Statistical significance was taken as *p* < 0.05. 

## 3. Results

The study included a total of 24 participants; 15 were female, with ages ranging between 19 and 41 years (mean age 26 ± 6.9 years). The average OSDI score was 14.58 ± 16.47. At the start, the blink rate was 25.4 ± 10.4/min; the TMH was 0.28 ± 0.11 mm; the TBUT was 10.7 ± −0.2; the MG grade upper lid score was 1.3 ± 0.7; and the MG grade lower lid grading was 2.1 ± 1.2. 

**Figure 3:**
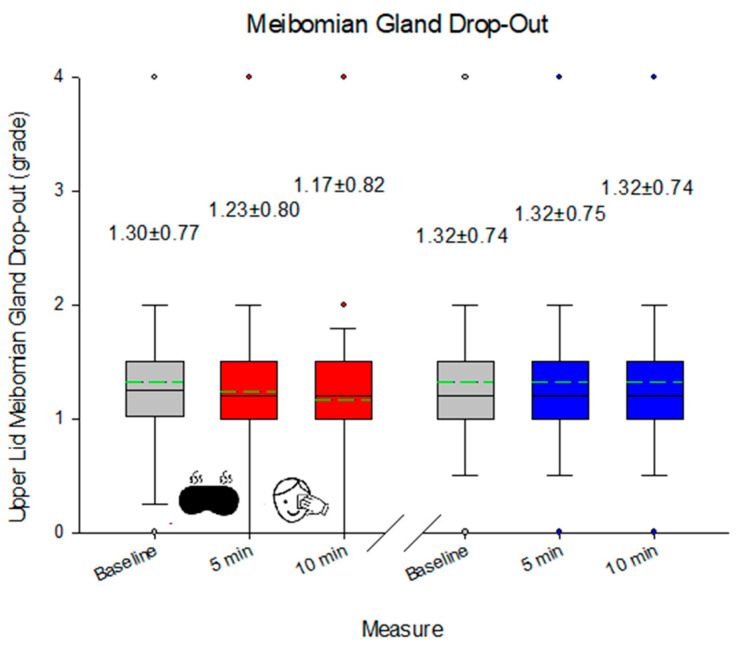
Meibomian gland dropout based on subjective analysis following eyelid warming and massage. Red indicates the impact of the heating/massage, whereas blue represents the eyelid inspection. The box indicates 25th/75th percentiles, whisker 95% confidence interval, and circular points to data beyond this, with the solid line representing the median value and the dashed line representing the mean.

**Figure 4:**
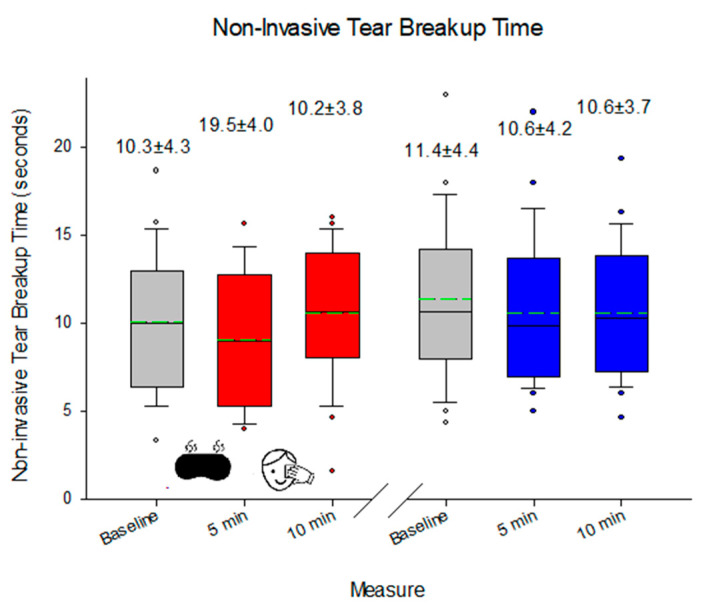
NIBUT before and after eyelid warming and massage. Red indicates the impact of the heating/massage and blue represents the eyelid inspection. The box indicates the 25th/75th percentiles, whisker 95% confidence interval, and circular points to data beyond this, with the solid line representing the median value and the dashed line representing the mean.

**Figure 5:**
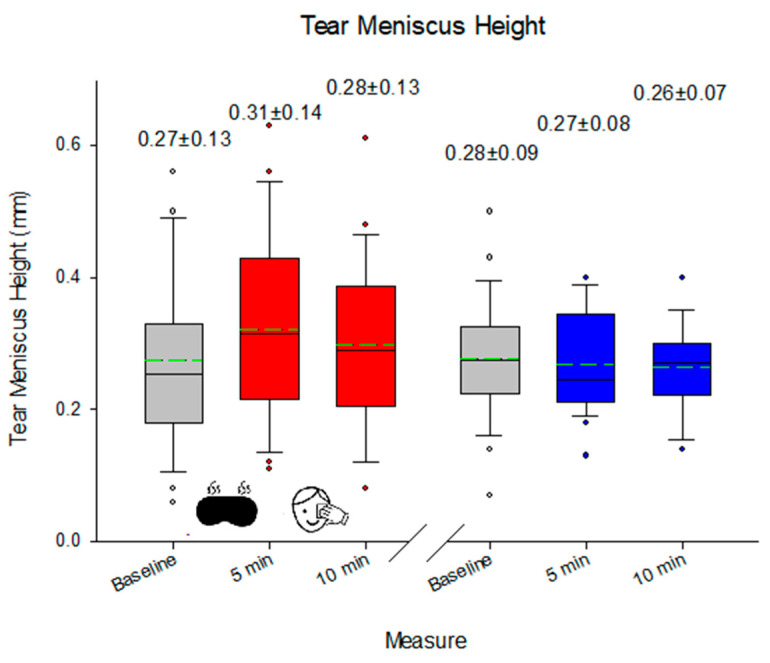
TMH before and after eyelid warming and massage. Red indicates the impact of the heating/massage and blue represents the eyelid inspection. The box indicates the 25th/75th percentiles, whisker 95% confidence interval, and circular points to data beyond this, with the solid line representing the median value and the dashed line representing the mean.

While there was no change in the appearance of the MGs of the lower lid in response to eyelid heating, eyelid massage, or multiple lid eversion (median 2.0, range 0.0 to 4.0; *p* = 0.782), there was a change in the appearance of the upper lid MGs 5 min after heating and lid massage (*p* = 0.025), but again, multiple lid eversion had no effect (*p* > 0.05; Figure 3). NIBUT decreased on second lid eversion (*p* = 0.049), although this was not evident on the third lid eversion (*p* = 0.090). The effect on NIBUT was also apparent with heating (*p* = 0.034 immediately after) but was sustained with 5 min of eyelid massage (*p* = 0.031; Figure 4). On the other hand, TMH increased with heating (*p* < 0.001), and this was sustained 5 min after eyelid massage (*p* = 0.011), but there was no lid eversion effect (*p* > 0.05; Figure 5). The blink rate was unaffected by heating, eyelid massage, or multiple eversions of the eyelids (median 24 blinks/min, range 8 to 59 blinks/min; *p* = 0.61). No adverse effect was reported during the study period.

## 4. Discussion

This study examined the effects of combined heating, lid massage, and lid eversion on MG imaging along with tear film parameters evaluation techniques to assess their association. The hypothesis was that the imaging technique uses infrared radiation to make the glands visible via their thermal signature, which is not the case with visible light. Hence, heating the eyelids and massaging them to increase the circulation of the oils within them prior to imaging could increase the visibility of the glands. This could be seen as a positive step to enhance imaging but could also cause additional variability with environmental conditions. Likewise, the study also examined whether repeated lid eversion could influence MG imaging. While it has previously been shown that multiple lid eversions cause significantly greater lid wiper epitheliopathy [21], this has not been examined for other tear film parameters. Likewise, the impact of heating and massage on MG visibility has not been previously explored for comparison with the results of this study. 

Eversion of the eyelids, performed three times 5 min apart, had no effect on subjectively graded MG imaging, along with tear meniscus height and blink rate. There was an increase in visibility of the glands of the upper lid (but only by a 0.1 grade reduction) 5 min after heating and massage, but no change in the appearance of the lower lid MGs was observed. The upper lid was in greater contact with the warm compress than the lower lid, so the process of lid eversion is likely to have a greater impact on the upper lid. This finding was accompanied by an increase in TMH and decrease in the NIBUT. The decrease in the NIBUT after heating the eyelid was similar to the reduction in the NIBUT that was observed after the second lid eversion with no heating or massage, although the effect was sustained after massaging. The increase in TMH could result from increased MG expression or decreased evaporation from an increase in lipid layer thickness, although it is unlikely to be due to reflex tearing, as multiple lid eversion has no effect on TMH. A similar increase in TMH was reported as a result of short-term wear of a warming moist chamber goggle for a video display terminal [22], including for patients who had refractive surgery [23]. In both instances, the likely cause was the prevention of tear evaporation due to enhanced stability of the lipid layer, a situation that may also apply to the current study.

Conversely, tear film stability decreased with heating and massage, but this seems to be due to disruption of the tear film due to the multiple lid eversion; why this was evident after the second lid eversion and not the third is not obvious. The reduced tear stability was sustained after heating and massaging, which may reflect the poor quality of the meibum lipids that were expressed [23]. 

Due to the limited time between repeat eyelid eversions (which is realistic for clinical practice, when the palpebral conjunctiva, meibography, and lid wiper epitheliopathy may be observed within an appointment), it was not possible to examine the lipid layer thickness and expressibility of the glands to fully understand the observed effects. In addition, these tests would have caused further disruption of the tear film which could have masked the effects. The sample size was limited, but the study was 95% powered to detect a 3.5 s change in NITBUT [24], 0.03 change in TMH, 3×/min change in blink rate [25], and 0.01 change in MG grade [26], taking a *p* < 0.05 as significant (G*Power v3.1.9.7, University of Dusseldorf, Dusseldorf, Germany).

## 5. Conclusions

Eyelid warming can increase the visibility of Meibomian glands, although this effect was only observed with upper lid imaging and the effect dissipated after 5 min of eyelid massage. The warming and massage also disrupted the tear film, as did multiple lid eversions, emphasising the previously stated need to use the least invasive tear film assessment techniques first [27]. 

## Data Availability

The research data were not all shared within the article; however they may be made available upon request.
